# Study on the Mechanical Properties of a Carbon-Fiber/Glass-Fiber Hybrid Foam Sandwich Structure

**DOI:** 10.3390/ma17092023

**Published:** 2024-04-26

**Authors:** Yingqiang Cai, Xiaolong Wang, Fenglin Ouyang, Qinglin Chen, Zhaoyi Zhu, Kuan Fan, Fan Ding

**Affiliations:** 1 School of Marine Engineeting, Jimei University, Xiamen 361021, China; wangxl2905@163.com (X.W.); 18897959348@163.com (F.O.); 1988zhuzhaoyi@163.com (Z.Z.); fankuan@jmu.edu.cn (K.F.); 15697311706@163.com (F.D.); 2 Ship and Ocean Engineering Key Laboratory of Fujian Province, Xiamen 361021, China

**Keywords:** yacht, FRP, composite materials, carbon–glass hybrid, sandwich structure, mechanical property

## Abstract

Considering the different structural strength requirements of different parts of fiberglass yachts, carbon fiber/glass fiber hybrid reinforcement can be applied to the skins of sandwich panels in special areas. This paper designs and prepares 12 foam sandwich panel samples composed of pure carbon fiber, a carbon fiber/glass fiber hybrid, pure glass fiber skin, and PVC and SAN foam sandwich, with reference to the layup structure of the outer panel of a fiberglass yacht. Through a comparative analysis of low-speed impact experiments, edge compression experiments, and short beam three-point bending experiments, we seek the optimal carbon fiber/glass fiber hybrid layup design scheme for local structures to guide production. The results show that a reasonable hybrid carbon fiber layup in fiberglass skin can effectively reduce the low-speed impact damage of the sandwich structure, reduce edge compression damage, and improve the bending and compression resistance of sandwich structure. The impact resistance, compression resistance, and shear resistance of the SAN sandwich structure are stronger than the PVC sandwich structure. The carbon fiber/glass fiber hybrid SAN foam sandwich structure can be used for the local structural reinforcement of special parts such as the bow, side, and main deck of fiberglass yachts.

## 1. Introduction

Sandwich composites, composed of high-strength skins and low-density core materials, have the advantages of being lightweight, high in strength and stiffness, and having a high design ability. They can reduce the self-weight of yachts, improve fuel efficiency and navigation performance [1], and are widely used in the structural design of composite material yachts, such as hulls, cabin walls, decks, hatches, etc. The skins often use fiber-reinforced composite laminates, which mainly bear axial bending and in-plane shear loads, while the shear loads in the thickness direction are borne by the core materials. Rigid foam materials have the advantages of being lightweight and high in strength, heat insulation, noise reduction, shock absorption, and energy absorption and are one of the most common forms of core materials. Commonly used foam core materials include polyvinyl chloride (PVC), styrene acrylonitrile (SAN), Polymethyl acrylamide (PMI), polyethylene terephthalate (PET), etc. PVC foam has an excellent stiffness and stiffness-to-weight ratio, SAN is a structural closed-cell thermoplastic core material, which has high toughness and impact-resistance characteristics, and has received more and more attention [2,3].

In recent years, researchers have conducted extensive studies on the mechanical behavior and damage failure of composite sandwich structures. Many scholars have focused on the low-speed impact damage and post-impact compressive performance of composite laminates and sandwich panels. Nin et al. [4] studied the damage and failure mechanisms of yacht composite laminates under low-speed impact and post-impact compression (CAI) conditions. They found that laying carbon fiber cloth in glass-fiber-reinforced plastic (GFRP) laminates can significantly improve impact resistance, and laying carbon fiber cloth in the first layer shows a better comprehensive mechanical performance. Fotouhi et al. [5] studied the mechanical properties of glass-fiber- and carbon-fiber-reinforced composite laminates under low-speed impact damage. They found that delamination is the main damage mode of the laminate, and the size of the damage depends on the fiber characteristics and layup sequence during the propagation stage. Caminero et al. [6] studied the impact damage tolerance of composite structures and studied the effects of layer thickness, layup sequence, and size on the performance of carbon-fiber-reinforced composite laminates through post-impact compression (CAI) tests. They found that the increase in impact energy reduces damage tolerance, while thick laminates have a higher CAI strength. Mohmmed et al. [7] used experimental and numerical methods to explore the low-speed impact performance and mechanical response of foam sandwich composites with different layup angles and evaluated the contact load history, peak load, and energy absorption at different impact energy levels. Yang et al. [8] studied the post-impact compressive performance of carbon fiber/epoxy resin panel PVC foam sandwich structures under extremely low temperature conditions. They found that low temperature seriously affects the post-impact compressive strength of composite sandwich structures, and the temperature effect on impact damage is greater than the performance of the material itself. Feng et al. [9] studied the low-speed impact response of foam core sandwich composites and found that the core density’s impact on the panel’s damage resistance is closely related to the lamination method of the panel skin. Peng et al. [10] studied the damage behavior and failure mechanism of large-size composite sandwich panels under low-speed impact and evaluated the effect of impact energy and structural parameters on it. Experiments and finite-element analysis showed that the local structural damage behavior varies with the impact energy, foam core height, and impact angle, while the layup sequence of the panel has a small impact on the impact resistance of the sandwich panel. The foam core plays a leading role in energy absorption, especially in the case of perforation damage. Chen et al. [11] developed a numerical model to predict the damage behavior of composite sandwich structures with honeycomb cores under low-speed perforation impact and experimentally verified the accuracy of the model. Tao et al. [12] designed a honeycomb (BIH) sandwich panel with lotus leaf veins as a biological prototype to improve the performance of the sandwich panel under low-speed impact. They established and effectively verified the finite-element (FE) model of the BIH sandwich panel using ABAQUS (https://www.3ds.com/products/simulia/abaqus) and systematically compared its mechanical performance with that of the conventional hexagonal honeycomb sandwich panel. The research results show that this structure can effectively improve the impact resistance of the BIH sandwich panel. Basha et al. [13] proved through low-speed impact and post-impact compression tests that CFRP/Kevlar sandwich composites have excellent damage resistance under impact and compressive loads. The damage at low impact energy is mainly matrix cracks and delamination, while at high-impact energy, the entire material exhibits fiber breakage.

Regarding the compressive performance of composite laminates and foam sandwich panels, many scholars have conducted extensive research. Wagih et al. [14] conducted a series of quasi-static indentation experiments on AS4D/TC350 carbon/epoxy resin laminate samples, finding that matrix cracking is actually the key damage mechanism, leading to a sudden loss of load capacity and gradual delamination. Higuchi et al. [15] studied the progressive damage in the open-hole compression (OHC) tests of composite laminates. From the experiment and simulation, it is clarified that the kink-band is initiated and propagated by the combined stress states consisting of longitudinal compression and in-plane shear around the intra-laminar cracks. Gunasegeran et al. [16] studied the energy absorption, load response, failure mode, and damage mechanics of sandwich structures composed of biomimetic polylactic acid (PLA) core and glass-fiber-reinforced polymer (GFRP) laminates under quasi-static indentation, finding that the energy absorption behavior of the biomimetic structure is largely influenced by its core geometric design. Waddar et al. [17] studied the buckling and dynamic response of sandwich beams composed of sisal fabric/epoxy and a synthetic foam core under compressive load through experiments and finite-element simulation. The results reveal that natural frequencies and the critical buckling load increase significantly with fly ash cenosphere content. Nieh et al. [18] studied the failure behavior of composite sandwich structures under compressive loading, especially the delamination of the panel and core layer, and pointed out that sandwich structures with thicker panels or shorter delamination lengths show higher buckling loads and greater failure strength. Mamalis et al. [19] conducted edge compression tests on eight different material combinations composed of four types of polymer foam cores (specifically PMI foam, two types of linear PVC foam, and polyurethane foam) and two types of FRP panel laminates, studying the compressive performance, collapse mode, and crushing characteristics of different types of composite sandwich panels. They focused on the collision energy absorption characteristics of the sandwich panel end crushing mode and analyzed the impact of material characteristics on the compressive response. Eyvazian et al. [20] studied the buckling and crushing behavior of foam core mixed composite sandwich columns under edge compression load, analyzing the effects of panel thickness, laminate sequence configuration, aspect ratio, boundary conditions, and thickness resin pin on the specimen behavior. The study shows that using resin pins can change the unstable Euler buckling mode to a more stable progressive end crushing, significantly increasing the buckling load, improving the bearing capacity and energy absorption capacity of the sandwich structure, and the opening has a significant impact on the stability of the laminate under compressive load [21].

Under bending conditions, composite laminates and foam sandwich panels can exhibit various failures. Betts et al. [22] conducted experimental studies on the mechanical behavior of sandwich beams made of bio-based fiber-reinforced polymers (FRP) sandwich panels and foam cores under three-point bending, observing three failure mechanisms, including top surface wrinkling/breaking, core shearing, and bottom surface tensile fracture. Farrokhabadi et al. [23] studied the mechanical behavior of multi-layer corrugated core composite sandwich panels under quasi-static three-point bending loading through experimental and numerical methods. The experimental results showed that the energy absorbed by the sandwich panel during loading and failure significantly increased, with the main damages including matrix cracking, fiber fracture, and delamination. Taghipoor et al. [24] studied the energy absorption and collapse behavior of a sandwich beam under a quasi-static three-point bending test, with its core being an expanded metal plate filled with rigid polyurethane foam. The research results showed that filling polyurethane foam and choosing the appropriate grid plate direction can improve the energy absorption capacity of the sandwich beam. Eyvazian et al. [25] studied the mechanical behavior of composite sandwich panels composed of PVC foam core and glass/epoxy panels under hemispherical indenter and three-point bending loading. By adjusting parameters such as the number, arrangement, and diameter of resin pins, they explored the effect of reinforcement parameters on the specimen. The results showed that using resin pins can significantly improve the maximum indentation load and bending strength, significantly improving the energy absorption capacity of the specimen. Hang et al. [26] conducted in-plane compression and four-point bending experiments and finite-element simulations on composite honeycomb sandwich structures with face-core delamination defects or impact damage to evaluate their sensitivity to damage. The research found that the structure’s sensitivity to impact damage is higher than its sensitivity to delamination defects. Mei et al. [27] prepared a composite X-core sandwich panel filled with foam using hot pressing technology to enhance the energy absorption capacity of the sandwich structure. They studied the bending behavior of hollow and mixed composite sandwich panels through three-point bending tests. The research showed that the load conditions have a significant impact on the fatigue performance of sandwich panels composed of polyurethane foam core and glass-fiber-reinforced polymer panels, with the fatigue life significantly reduced under full reverse load conditions, about 10% of the no-load conditions [28].

Some scholars choose to add matrix materials or design new structures to improve the mechanical properties of composites. Liu et al. [29] improved the interlaminar properties of GF/EP laminate composites by toughening the epoxy resin (EP) matrix with the addition of soft carboxyl-terminated nitrile (CTBN) rubber particles and rigid nano-SiO2. Kerche et al. [30] designed and manufactured a Z-pin-reinforced foam core sandwich panel and determined the optimal pin position, angle, and parameter values through a search algorithm, increasing the bending stiffness of the sandwich panel. Yang et al. [31] proposed a new petal triangular core sandwich panel (SP-PSC), and improved the negative Poisson’s ratio effect by changing the panel material to unidirectional CFRP. Yalkin et al. [32] used perforated foam and perforated sutured foam as the core material to improve the mechanical properties of foam core sandwich composites, while maintaining a light weight and high strength. Dimassi et al. [33] inserted carbon fiber and glass fiber dry bundles into a poly(methyl methacrylate) foam core and enhanced the compressive strength and stiffness of the core through specific tilt angles and pin patterns. Kaya et al. [34] studied the impact resistance of Z-pin-reinforced sandwich composites and pointed out that the type of Z-pin, distribution density, and the adhesion between the Z-pin and panel material will have a significant impact on the impact performance of composite sandwich structures. After inserting glass or carbon fiber pins, the bending load, strength, and modulus of the sandwich composite material significantly increased [35]. Sharaf et al. [36] used a new type of thermal insulation sandwich panel with a glass fiber shell for the cladding of buildings. It is composed of GFRP panels, ribs, and a soft polyurethane foam core. These panels are designed to resist wind loads and can provide an excellent thermal insulation performance, providing a reference for the thermal insulation of composite material boats.

In conclusion, in the existing literature, the research on fiber-reinforced composites mainly focuses on the following aspects: (1) the impact of in-plane or interlayer hybrid weaving structure on the mechanical properties of composites, (2) the study of the mechanical properties of single fiber composite laminates or sandwich structures, and (3) the study of the mechanical properties of hybrid fiber composite laminates. However, when considering the combination of hybrid fiber skins and foam core materials, there is relatively little research on the mechanical properties of carbon fiber/glass fiber hybrid foam sandwich structures. The mechanical performance differences of composite structures composed of different skins and core materials are significant. Based on cost considerations and lightweight requirements, yacht structures usually adopt a sandwich structure of fiberglass skin and foam core material. In extreme conditions, there is a risk of structural failure in key parts such as the bow and side of the yacht. Local reinforcement with carbon fiber/glass fiber hybrid can effectively improve the structural strength requirements of different parts of the yacht. This paper designs and prepares 12 kinds of foam sandwich board samples composed of pure carbon fiber, carbon fiber/glass fiber hybrid, pure glass fiber skin, and a PVC/SAN foam core. Based on the ASTM standards, the performance characteristics of the samples under typical conditions such as compression, bending, and low-speed impact are studied to improve the sandwich structure design of fiberglass yachts and enhance the safety and reliability of yacht structures.

## 2. Experimental Design

### 2.1. Experimental Materials

**Figure 1:**
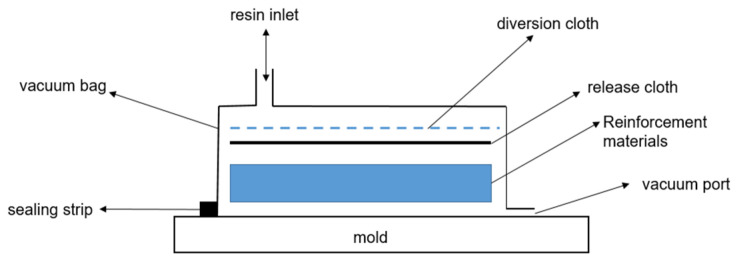
Schematic diagram of the vacuum adsorption process.

Based on a certain fiberglass yacht hull outer panel sandwich structure scheme, 12 schemes of pure glass-fiber, glass-fiber/carbon-fiber hybrid, pure carbon-fiber skin, and a PVC/SAN foam sandwich structure were designed, as shown in Table 1 and Table 2, to compare the impact of fiber hybrid and foam sandwich on the mechanical properties of the composite material sandwich structure. Among them, CSM300 is a glass fiber short-cut mat with a single layer thickness of 0.7 mm; 800EGBM and 600EGBM are glass fiber composite mats with laying angles of −45°/+45°, and the single layer thicknesses are 0.8 mm and 0.6 mm, respectively; BC300 is a carbon fiber cloth with a laying angle of 0°/90°, and the single layer thickness is 0.25 mm; the thicknesses of PVC80 foam and SAN80 foam are both 15 mm. They are prepared by the vacuum adsorption molding method, as shown in Figure 1. The process is as follows: Spread the “dry” reinforcement material (glass fiber/carbon fiber, foam core, etc.), release cloth and diversion cloth on the mold, then seal it with a vacuum bag and sealing strip, and extract the air from the vacuum bag. Negative pressure is formed in the mold, and the unsaturated resin is pressed into the fiber layup through the pre-laid pipes under the action of air pressure, so that the resin fully infiltrates the reinforced material and eventually fills the entire mold. After the product has cured, remove the vacuum bag material and remove it from the mold. Obtain the products you need. Due to the large number of samples required for the experiment, large-size sample plates are prepared first, and then the samples are made using a cutting machine according to the requirements of the ASTM D7136 standard [37] (i.e., the test method for measuring the impact damage resistance of fiber-reinforced polymer matrix composites), the ASTM C364 standard [38] (i.e., the standard test method for the side pressure strength of sandwich structures), and the ASTM C393 standard [39] (i.e., the standard test method for the core shear performance of beam bending sandwich structures). The average thickness of the samples is shown in Table 1 and Table 2, and the size of the samples is shown in Table 3.

### 2.2. Testing and Characterization

(1) Low-Speed Impact Test

Low-speed impact tests mainly include drop hammer impact, pendulum impact, and ballistic impact. Due to the simplicity and low cost of the drop hammer impact principle, it can better simulate low-energy impact. To simulate the damage situation of the yacht structure under low-speed impact from external loads, a fully automatic drop hammer impact testing machine (WCJ-1500AD, Shanghai Hualong Test Instrument Co., LTD, Shanghai, China) is used to conduct low-speed impact tests on the aforementioned 12 sandwich structures in accordance with the ASTM D7136 standard [37]. This method employs a drop hammer apparatus equipped with a hemispherical impact head to vertically strike a rectangular plate, subjecting it to an out-of-plane concentrated impact load. The samples are fixed by four special fixtures, with a clamping force of 1000 N. The experimental impact head is hemispherical, with a diameter of 10 mm and a mass of 5.7 kg, and the impact head is equipped with a pressure sensor with a sampling frequency of 200 kHz. By adjusting the height of the impact head, different impact speeds and energies can be simulated, up to a maximum of 3 m. It is equipped with a pneumatic anti-secondary impact device to ensure that the impact head does not rebound and cause secondary impact damage.

According to the navigation conditions of fiberglass yachts, five kinds of impact energies, 10 J, 15 J, 20 J, 25 J, and 30 J, are selected to conduct low-speed impact tests on sandwich panel samples. The samples are freely impacted from a set height at the geometric center of the skin on the samples, and the impact force and impact speed during the test are recorded. Then, the impact energy, impact head displacement, and absorbed energy can be calculated according to Formulas (1)–(3).

(1) $$E_{i}=\frac{mv_{i}^{2}}{2}$$

where $E_{i}$ is the measured impact energy, J; *m* is the mass of impactor, kg.

(2) $$\delta \left(t\right)=\delta_{i}+v_{i}t+\frac{gt^{2}}{2}-\int_{0}^{t}\left(\frac{F\left(t\right)}{m}dt\right)dt$$

where $\delta \left(t\right)$ is the impactor displacement at time *t*, *m*; $\delta_{i}$ is the impactor displacement from the reference location at time *t* = 0, m.

(3) $$E_{a}\left(t\right)=\frac{m(v_{i}^{2}-{v\left(t\right)}^{2})}{2}+mg\delta \left(t\right)$$

where $E_{a}\left(t\right)$ is the absorbed energy at time *t*, J.

(2) Edge Compression Test

**Figure 2:**
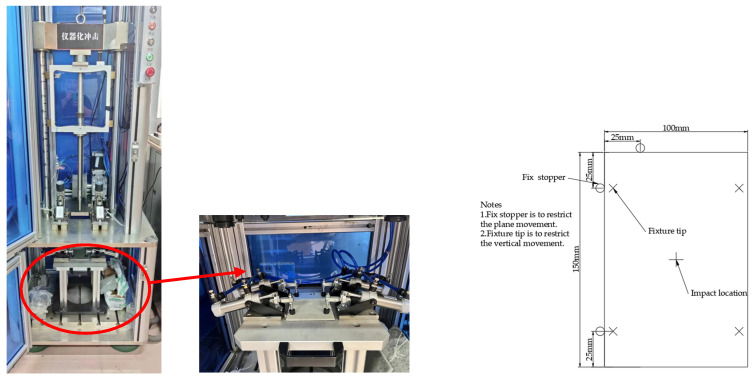
Drop weight impact test.

The edge compressive strength of the sandwich structure of the yacht’s outer panel directly affects the stability and reliability of the overall structure. To compare the compressive capacity of the aforementioned 12 sandwich structures, a universal testing machine (WDW-100C, Shanghai Hualong Test Instrument Co., LTD, Shanghai, China) is used to conduct the structural edge compressive strength test of the composite material sandwich panel in accordance with the ASTM C364 standard [38]. The test uses standard fixtures, and the skin near the loading end is laterally supported by clamping, and a downward force perpendicular to the sandwich panel is applied until the sample fails, as shown in Figure 2. The test range of the testing machine is 0~100 KN, the loading speed is 0.5 mm/min, and the load–displacement data are recorded in real time through the data acquisition system of the testing machine. The stress of the sample skin can be calculated according to Formula (4):

(4) $$\sigma_{compress}=\frac{P}{A}$$

where $\sigma_{compress}$ is the compressive stress, MPa; *P* is the ultimate load, N; *A* is the area of the two skins, which can be calculated by multiplying the thickness of the upper and lower skins by the width.

(3) Short Beam Three-Point Bending Test

**Figure 3:**
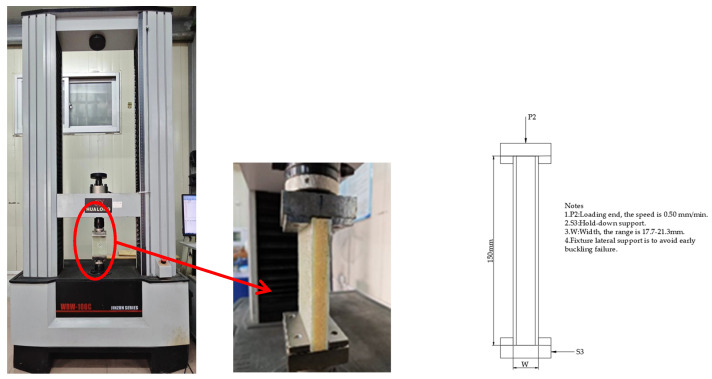
Edge compression test.

When a composite sandwich structure is subjected to bending loads, it may experience skin panel failure, core material failure, and interface failure between the skin and the core material. At this point, the bending moment will be converted into tensile stress and compressive stress within the upper and lower skins, and the core material mainly bears shear stress. In order to achieve core material failure under bending loads, a shorter support span and three-point bending loading method are usually used. This can minimize the bending stress in the upper and lower skins, and first cause core material shear failure or local core material compression failure at the center load point. A universal testing machine (WDW-100C) is used according to the ASTM C393 standard [39] to perform bending loading with a three-point standard configuration. The sample size is shown in Table 3, the bottom support span is 150 mm, and the upper part is centrally loaded, as shown in Figure 3. The loading speed is 6 mm/min until the material reaches its limit strength. The load–displacement data are recorded in real time through the data acquisition system of the testing machine. According to Formula (5), the limit shear strength of the sample core material can be calculated. It can evaluate whether the core material shear failure occurs before the skin failure; whether it is core material shear failure or interface debonding failure between the core material and the skin, both are effective forms of failure.

(5) $$F_{s}^{ult}=\frac{P_{max}}{\left(d+c\right)b}$$

where $F_{s}^{ult}$ is the shear ultimate strength, MPa; $P_{max}$ is the maximum force prior to failure, N; d is the sandwich thickness, mm; c is the core thickness, mm; b is the sandwich width, mm.

## 3. Results and Analysis

### 3.1. Low-Speed Impact Test

Low-speed impact loads can cause internal damage to the skins of composite sandwich panels. This damage primarily includes interlayer delamination of the skin, fiber fracture, and matrix cracking. It is often difficult to accurately assess this damage with the naked eye. To accurately evaluate the damage, it is necessary to use ultrasonic C-scan technology to analyze the extent of damage to the internal defects of the impact sample skins. In this study, a portable ultrasonic detection device (PHASCAN300 16/64, Guangzhou Botech Testing Instrument Co. Ltd., Guangzhou, China) is used, which is equipped with a 2.25 MHz probe with 64 linearly arranged crystal elements. The probe model is 2.25L64. In conjunction with the delay wedge SB26-N0L-IH and a wheel scanner, the surface of the sample is scanned. Based on the displacement relationship between the probe and the wheel, a C-scan image can be generated. This method allows for a more precise evaluation of the internal damage to the composite material.

**Figure 4:**
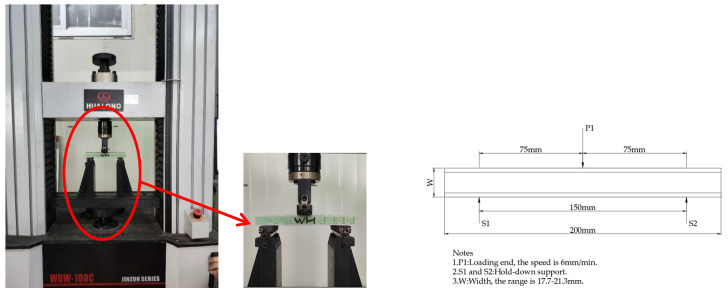
Short beam three-point bending test.

**Figure 5:**
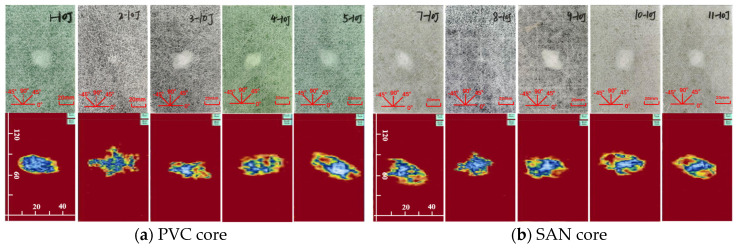
Physical picture of 10 J impact damage and ultrasonic C-scan image.

**Figure 6:**
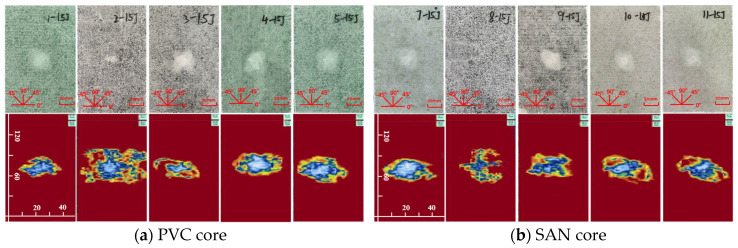
Physical picture of 15 J impact damage and ultrasonic C-scan image.

**Figure 7:**
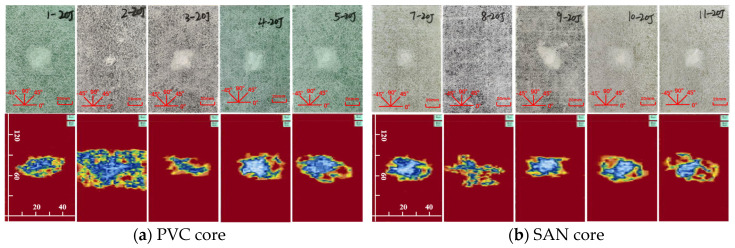
Physical picture of 20 J impact damage and ultrasonic C-scan image.

**Figure 8:**
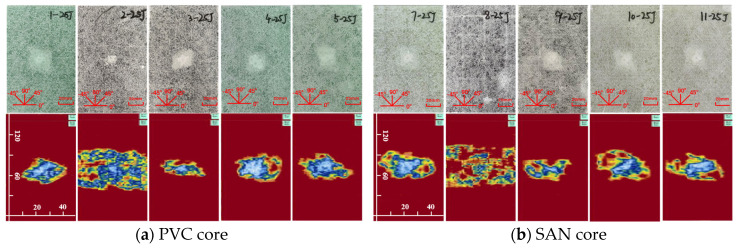
Physical picture of 25 J impact damage and ultrasonic C-scan image.

The impact damage caused by five kinds of impact energy (10 J, 15 J, 20 J, 25 J, 30 J) can be determined by the surface damage and ultrasonic C-scan images to identify the damage mode, and then analyze the low-speed impact resistance of sandwich panels with different layup structures. The impact surface damage and ultrasonic C-scan results are shown in Figure 4, Figure 5, Figure 6, Figure 7 and Figure 8. Due to the poor impact resistance of the pure carbon fiber laminate skin, obvious damage has already occurred under the impact energy of 15 J. Therefore, the ultrasonic C-scan only compares the sandwich structures 1–5 and 7–11.

Under the impact energy of 10 J, the overall surface of the skin on the sample is smooth without pits. Scheme 8 has the least damage, and there is no obvious damage to the surface. However, the ultrasonic C-scan shows that different degrees of damage have occurred inside the sample, and the impact damage area is basically elliptical, mainly delamination damage, with a maximum of only 8 × 10 mm. Under the impact energy of 15 J, slight impact pits appear on the surface of the skin on the sample. Scheme 2 has a 13 mm horizontal fiber area of damage, Scheme 8 has no obvious visual damage, and other schemes have ±45° fiber damage varying from 20 to 25 mm. Under the impact energy of 20–25 J, matrix cracking, fiber fracture, and cross-damage along the fiber direction of ±45°, 0°, and 90° successively appear in the impact point area, and the impact area significantly increases. Under the impact energy of 30 J, the damage range continues to expand, and the impact energy is mainly absorbed by the upper skin and foam core. The energy absorption mainly presents in the form of fiber fracture, and the carbon fiber layup mixed in the lower skin does not significantly improve the low-speed impact resistance of the sandwich structure.

**Figure 9:**
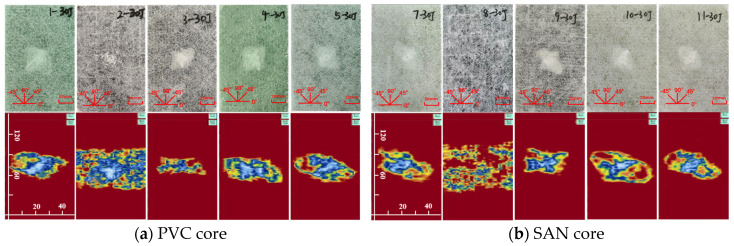
Physical picture of 30 J impact damage and ultrasonic C-scan image.

It is worth noting that although Scheme 2 has less visible surface damage, the ultrasonic C-scan shows that its internal damage is larger, and this phenomenon is more obvious in the 20–30 J impact. Obviously, the PVC sandwich structure with carbon fiber layup arranged at the top of the upper skin is not ideal in terms of impact resistance, and this layup structure design scheme is not suitable for use in the parts of the ship structure that are susceptible to impact. Under various impact energies, Scheme 8 is almost undamaged visually, and the ultrasonic C-scan image shows that its damage is also smaller, and only large-area damage appears when the impact energy is 30 J (Figure 8b). This shows that not only the fiber material, but also the core material has an important impact on the impact resistance of the sandwich structure. Although carbon fiber has a high strength, its fracture toughness and ductility are poor, which leads to greater brittleness of the structure, while the SAN foam core has the characteristics of high toughness and impact resistance, and the combination of the two has a positive effect. Comparing “Subfigure a of Figure 5, Figure 6, Figure 7, Figure 8 and Figure 9” and “subfigure b of Figure 5, Figure 6, Figure 7, Figure 8 and Figure 9”, under the same layup parameters, the impact resistance of the SAN foam sandwich panel is better than that of the PVC sandwich panel, and the impact damage is smaller. The ultrasonic C-scan image shows that the damage of Scheme 3 and Scheme 9 under various energy impacts is also relatively small, which shows that the mixed carbon fiber layup at the bottom of the upper skin also has a significant improvement in impact resistance.

**Figure 10:**
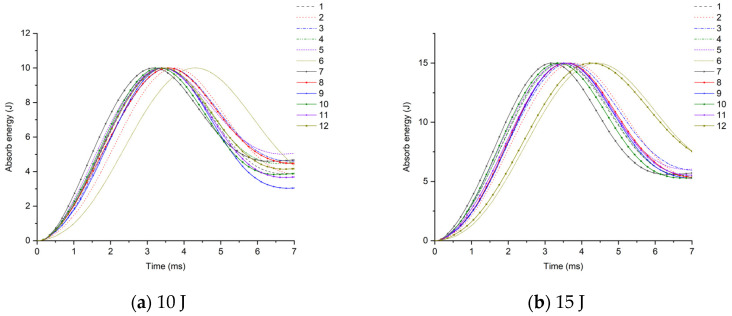

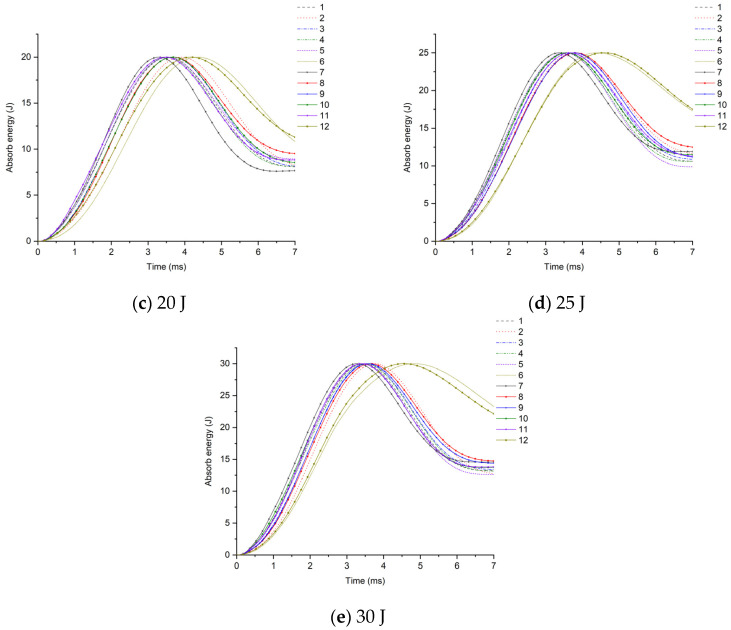
Sample impact energy absorption–time curve.

Further analysis of the impact resistance of different structural scheme samples is conducted by combining the energy absorption–time history and contact force–time history of the samples under low-speed impact. As shown in Figure 10, under the impact energy of 10 J, the energy absorption of the glass–carbon hybrid sandwich panel is the lowest, and the impact resistance is the best. Under the impact energy of 15 J, except for the pure carbon fiber scheme, the energy absorption rates of other schemes tend to be concentrated, and the difference is not obvious. Under the impact energy of 20 J–30 J, the energy absorption of Scheme 3 and Scheme 9 is the lowest. When the skin parameters are the same, the energy absorption of the SAN foam sandwich panel is significantly lower than that of the PVC foam sandwich panel, indicating that the internal damage is relatively small, which is basically consistent with the results of the ultrasonic C-scan image.

**Figure 11:**
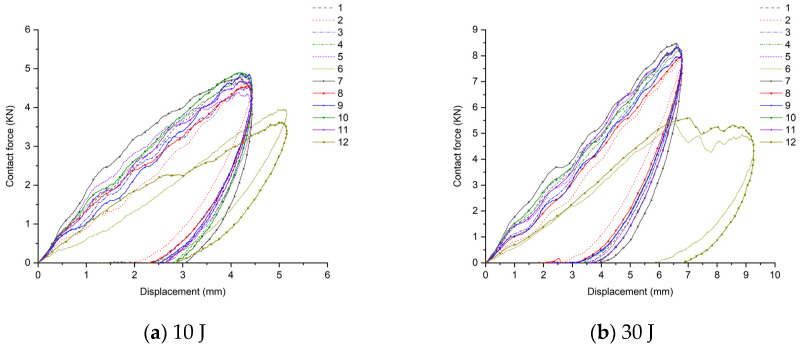
Contact force–displacement curve after the sample is impacted.

The contact force–displacement curve of the sample is shown in Figure 11, and the peak contact forces of the 12 scheme samples under five kinds of impact energy are shown in Table 4. After the sample is hit by the impact head, as the deformation increases, the contact force basically shows a linear growth. The larger the slope, the higher the peak contact force, and it increases with the increase in impact energy (as shown in Table 4). The area enclosed by the contact force–displacement curve and the X-axis is the absorbed energy of the sample. Its numerical size is basically consistent with Figure 9. The more energy absorbed, the greater the damage. According to the experimental data in Table 1, under the same skin conditions, the peak contact force of the SAN sandwich panel is greater than that of the PVC sandwich panel. And the peak contact force of the glass–carbon hybrid sandwich panel is smaller than that of the pure glass fiber sandwich panel, and larger than that of the pure carbon fiber sandwich, showing a clear hybrid effect.

In conclusion, the comparative analysis from multiple dimensions such as the surface damage of the specimen under low-speed impact, ultrasonic C-scan images, energy absorption rate, and peak contact force indicates that in areas prone to impact loads such as the bow, side, and main deck, it is advisable to intersperse carbon fiber layers in the upper skin facing the impact. When using SAN core material, the carbon fiber layer can be placed at the top of the upper skin, and when using PVC core material, the carbon fiber can be placed at the bottom of the upper skin. In terms of improving the impact resistance of the sandwich structure, the role of mixing carbon fiber layers in the lower skin is not significant. However, under bending conditions, it can enhance the hull’s ability to resist bending deformation.

### 3.2. Edge Compression Test

**Figure 12:**
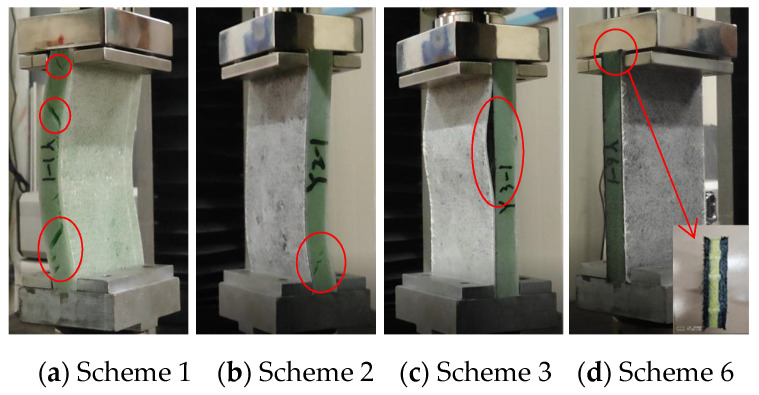
Compression failure mode of the PVC sandwich panel.

**Figure 13:**
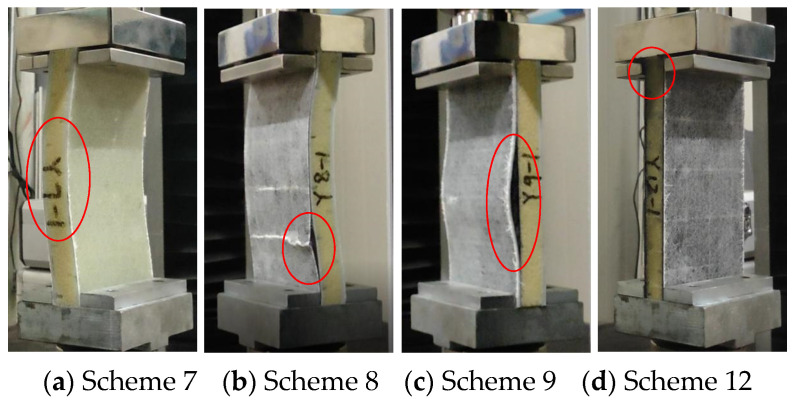
Compression failure mode of the SAN sandwich panel.

**Figure 14:**
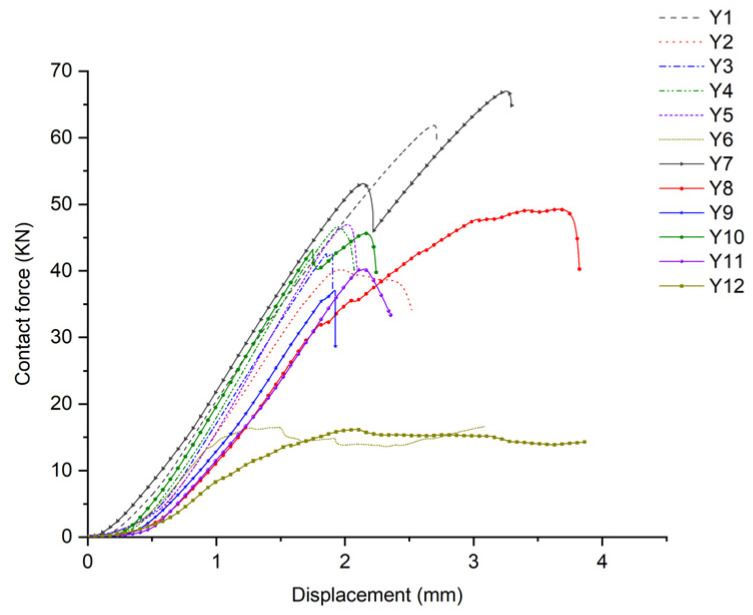
Contact force–time curve under edge compression conditions.

According to the ASTM C364 standard [38], the loading speed of the edge compression test is set to 0.5 mm/min. When the contact force–displacement curve suddenly drops or directly interrupts, it is considered to be a structural failure. The forms of sandwich panel compression failure are shown in Figure 12 and Figure 13, and the contact force–displacement curve is shown in Figure 14. Three types of failure modes occurred in the edge compression test of the composite sandwich panel. The first type is the overall compression bending of the sandwich panel and the shear failure of the foam core material leading to structural failure, as shown in Figure 12a,b and Figure 13a,b. During the compression process, the upper skin shows compression buckling, the lower skin shows tensile buckling, and the core material inside shows a 45° shear failure characteristic, but the skin and core material have not been dislocated, and the interface has not been damaged. The second type is the buckling after the skin and core material are debonded, leading to structural failure, as shown in Figure 12c and Figure 13c. When there is a large difference in the compression deformation rate between the skin and the core material, the interface of the skin and the core material moves and then causes debonding. Whether it is the overall bending of the specimen or the buckling of the skin, the bending direction is always towards the skin containing the carbon fiber layer. The third situation is the end crushing of the sandwich panel, as shown in Figure 12d and Figure 13d. This form of structural failure only occurs in the compression test of pure carbon fiber board specimens. During the compression process, the specimen did not show obvious bending deformation and damage, the upper and lower ends of the specimen appeared crushing and burring, the burrs were stuck in the fixture, and the foam core material produced permanent plastic deformation.

**Figure 15:**
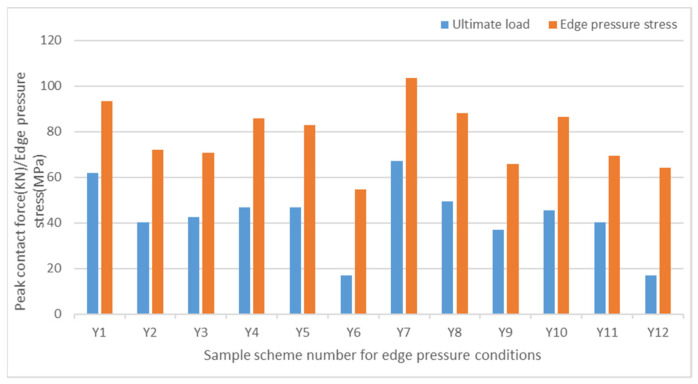
Peak contact force and stress peak under edge compression conditions.

From the failure modes of sandwich panel compression, it can be seen that the failure modes of SAN foam sandwich panels and PVC foam sandwich panels are basically similar under edge compression conditions. However, in terms of compression resistance, SAN foam is significantly superior to PVC foam. The resistance to deformation of the compressive load of the SAN sandwich structure is not obvious, and there is no obvious fracture of the foam core material after structural failure, but it appears in the form of foam densification and permanent deformation. As can be seen from Figure 14 and Figure 15, the edge compression failure modes and load-bearing levels of different layup structures are quite different, and the debonding of the skin and core material will significantly reduce the structural strength and stiffness of the sandwich panel. During the experiment, after the specimen reached the peak contact force, the contact force dropped sharply, but the sandwich panel still retained some stiffness and would not completely collapse. The pure carbon fiber sandwich panel shows an increase in displacement while the load remains essentially unchanged due to the occurrence of end crushing. This results in a horizontal curve, with the load level being relatively low but the stress peak located in the middle level. The experimental data show that the compression resistance of carbon fiber–glass fiber hybrid sandwich panels is weaker than that of pure glass fiber sandwich panels, and the bending resistance has also shown degradation. Although the laminated strength of carbon fiber and glass fiber materials does not differ significantly, when the number of layers is the same, the thickness of the glass fiber laminate is significantly greater than that of the carbon fiber laminate. This is the reason why the compression resistance of the pure glass fiber sandwich panel is stronger in the experiment, and it is also the reason why the sample always bends towards the skin direction containing the carbon fiber layer. Therefore, in the structural design of the sandwich panel, the layers of the upper and lower skins should be arranged symmetrically as much as possible to avoid the degradation of bending resistance.

It is worth noting that sandwich panels with carbon fiber mixed at the bottom of the skin are more prone to skin debonding damage (Figure 12c and Figure 13c). The bonding force between the carbon fiber layer and the foam core material is obviously weaker than the glass fiber layer. To avoid the debonding failure of the skin and core material under edge compression conditions, the carbon fiber layer should be placed at the top or middle of the skin as much as possible, and the glass fiber layer should be placed at the bottom of the skin.

### 3.3. Short Beam Three-Point Bending Test

**Figure 16:**
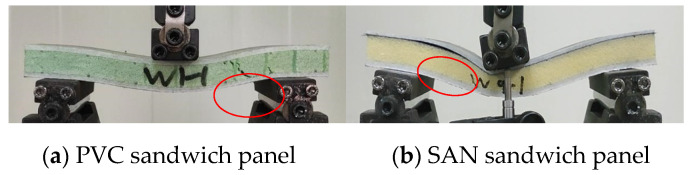
Short beam three-point bending sample failure.

According to the ASTM C393 standard [39], a short beam three-point bending test is used. This test minimizes the influence of bending stress in the skin under bending load, and the specimen undergoes shear failure or compression failure of the core material to test the shear failure resistance of the sandwich panel. The experimental results show that under the condition of short beam three-point bending, the specimen mainly has two forms of failure, shear failure of the core material and debonding of the upper skin and core material, as shown in Figure 16. Compared with fiber-reinforced materials, the strength and stiffness of the matrix and foam core material are much weaker, and they often fail first when subjected to load [40]. When the load exceeds the shear strength of the foam, the foam core material shows shear failure in the 45° direction. For most composite materials, their compressive strength is lower than their tensile strength, and the upper skin is usually subject to compressive failure. The experimental results also confirm this point, and there is a clear fiber fracture sound when the upper skin is compressed and damaged. It is worth noting that, unlike PVC sandwich panels, the structural failure of SAN sandwich panels starts from the debonding of the skin and core material. Before failure, there is no obvious sign of foam shear failure. It is due to the large local deflection deformation that causes the local compressive stress to concentrate, which eventually leads to structural failure [41].

In terms of overall bending performance, the bending strength is best when the upper and lower contact surfaces of the foam are carbon fiber layers. The bending performance of SAN foam sandwich panels is superior to PVC foam sandwich panels. However, PVC foam can recover to its original state after structural failure. After SAN foam bears a larger compressive load, although there are fewer foam fragmentation phenomena, the foam permanently densifies and cannot recover to its original state.

**Figure 17:**
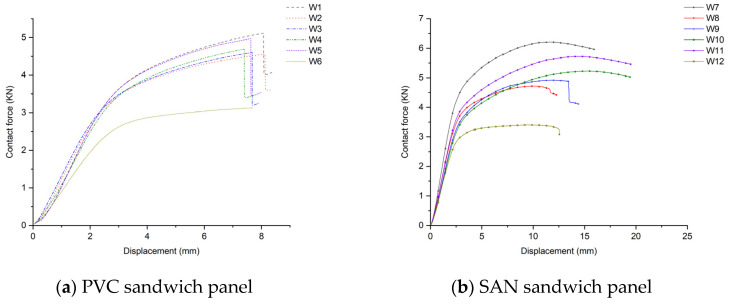
Contact force–displacement curve in a short beam three-point bending test.

During the experimental loading process, the contact force–displacement curve shows linear characteristics when the load is small. As the load increases, the slope gradually decreases, showing that the specimen changes from elastic bending deformation to yield failure, and finally the structure is damaged and the load-bearing capacity decreases, as shown in Figure 17. The limit shear stress data of the core material are shown in Table 5. The experiment also found that both PVC and SAN foams have a good buffer toughness. As long as the load does not exceed their plastic limit, they can rebound and recover after unloading. The bending resistance performance varies among different layer structures. Among them, the performance of pure glass fiber boards is the best and can withstand larger bending loads. After replacing part of the glass fiber layer in the upper skin with a carbon fiber layer, the bending performance degrades to a certain extent, and the bending performance after corresponding replacement in the lower skin does not change much. The main reason is that the upper skin is the compressed surface and the lower skin is the tensile surface. The compressive strength is much lower than the tensile strength. At this time, the thickness of the skin laminate becomes the main influencing factor. Therefore, the thickness of the upper and lower skins should be basically the same to avoid the degradation of bending performance.

It is worth noting that when the PVC sandwich panel reaches the peak contact force, the foam undergoes shear failure, and the load-bearing capacity drops sharply. In contrast, the SAN sandwich panel shows local deflection deformation and local compression contact stress concentration, leading to structural failure and obvious buckling deformation. Its curve changes slowly, and the load-bearing capacity shows a slow downward trend. The experimental data in Table 5 also show that the limit shear strength of the SAN foam sandwich panel is better. It can withstand larger deflection deformation and bending stress, and it will not suddenly fail under harsh conditions.

## 4. Conclusions

(1) When the skin layup parameters are the same, the peak contact force of the SAN sandwich panel under low-speed impact conditions is greater than that of the PVC sandwich panel, the energy absorption is significantly lower than that of the PVC sandwich, and the impact resistance is better than that of the PVC sandwich panel, with less impact damage. The peak contact force of the glass–carbon hybrid sandwich panel is smaller than that of the pure glass fiber sandwich panel, and larger than that of the pure carbon fiber sandwich, showing a clear hybrid effect. Mixing carbon fiber layers in the upper skin of the impact surface can significantly improve the impact resistance of the sandwich panel, with the impact resistance improving by 5.13%. While intermixing carbon fiber layers in the lower skin does not improve much, but it can enhance the ability of the hull to resist bending deformation under bending conditions.

(2) Under extreme edge compression conditions, sandwich panels will suffer structural failures such as core material shear failure, skin core material debonding, and end crushing, which will significantly reduce the strength and stiffness of the hull structure. The use of SAN foam core material can significantly enhance the compressive performance of the sandwich panel. The compressive performance of the original layup is improved by 10.7%, and it resists load in the form of compression densification and permanent deformation. The compression resistance of carbon fiber and glass fiber hybrid sandwich panels is weaker than that of pure glass fiber sandwich panels, and the bending resistance decreases to varying degrees. Therefore, in the structural design of sandwich panels, the upper and lower skin layers should be arranged as symmetrically as possible to avoid the degradation of bending resistance. And the carbon fiber layup should be placed on the top or middle of the skin as much as possible to avoid debonding of the skin and core material.

(3) Under the condition of three-point bending of short beams, sandwich panels mainly have two forms of failure: core shear failure and upper skin delamination from the core. The former mainly occurs in PVC sandwich panels, where the load-bearing capacity will drop sharply after the load reaches its peak; the latter mainly occurs in SAN sandwich panels, where there is no obvious buckling deformation when the structure fails after reaching the load limit, and the load-bearing capacity shows a slow downward trend. The ultimate shear strength of the SAN foam sandwich panel is higher, which allows it to withstand greater deflection deformation and bending stress. The ultimate shear stress has increased by 24.32%.

## Figures and Tables

**Table 1:** Structural parameters of PVC sandwich panel samples.

Scheme Number	1	2	3	4	5	6
Upper skin layup	CSM300	CSM300	CSM300	CSM300	CSM300	CSM300
	800EGBM	BC300	800EGBM	800EGBM	800EGBM	BC300
	800EGBM	BC300	800EGBM	800EGBM	800EGBM	BC300
	600EGBM	600EGBM	BC300	600EGBM	600EGBM	BC300
	600EGBM	600EGBM	BC300	600EGBM	600EGBM	BC300
Core material	PVC80	PVC80	PVC80	PVC80	PVC80	PVC80
Lower skin layup	800EGBM	800EGBM	800EGBM	BC300	800EGBM	BC300
	800EGBM	800EGBM	800EGBM	BC300	800EGBM	BC300
	600EGBM	600EGBM	600EGBM	600EGBM	BC300	BC300
	600EGBM	600EGBM	600EGBM	600EGBM	BC300	BC300
Thickness/mm	21.3	20.2	20.6	20.2	20.6	17.7

**Table 2:** SAN sandwich panel sample structural parameters.

Scheme Number	7	8	9	10	11	12
Upper skin Layup	CSM300	CSM300	CSM300	CSM300	CSM300	CSM300
	800EGBM	BC300	800EGBM	800EGBM	800EGBM	BC300
	800EGBM	BC300	800EGBM	800EGBM	800EGBM	BC300
	600EGBM	600EGBM	BC300	600EGBM	600EGBM	BC300
	600EGBM	600EGBM	BC300	600EGBM	600EGBM	BC300
Core material	SAN80	SAN80	SAN80	SAN80	SAN80	SAN80
Lower skin layup	800EGBM	800EGBM	800EGBM	BC300	800EGBM	BC300
	800EGBM	800EGBM	800EGBM	BC300	800EGBM	BC300
	600EGBM	600EGBM	600EGBM	600EGBM	BC300	BC300
	600EGBM	600EGBM	600EGBM	600EGBM	BC300	BC300
Thickness/mm	21.3	20.2	20.6	20.2	20.6	17.7

**Table 3:** Sample geometric dimensions unit: mm.

Name	Preparation Size	Impact Sample Size	Compression Sample Size	Bending Sample Size
L × W	800 × 550	150 × 100	150 × 100	200 × 75

**Table 4:** Peak contact force after the sample is impacted unit: KN.

Scheme Numbers	1	2	3	4	5	6	7	8	9	10	11	12
10 J	4.81	4.68	4.53	4.68	4.38	3.96	4.78	4.57	4.88	4.90	4.68	3.62
15 J	5.86	5.68	5.65	5.85	5.64	4.86	5.92	5.45	5.94	5.93	5.93	4.93
20 J	6.62	6.45	6.68	6.71	6.48	5.48	7.36	6.36	6.62	6.96	6.44	5.19
25 J	7.41	7.12	7.43	7.56	7.45	6.03	7.99	7.25	7.48	7.65	7.47	5.73
30 J	8.39	7.96	8.33	8.17	8.34	5.38	8.48	7.97	7.96	8.33	8.34	5.61

**Table 5:** Limit shear stress of core material unit: MPa.

Scheme Numbers	1	2	3	4	5	6	7	8	9	10	11	12
Shear stress	1.85	1.70	1.75	1.80	1.91	1.31	2.30	1.80	1.86	1.87	2.18	1.36

## Data Availability

Data are contained within the article.
